# CaO as Drop-In Colloidal Catalysts for the Synthesis of Higher Polyglycerols

**DOI:** 10.1002/chem.201405906

**Published:** 2015-02-13

**Authors:** Fiona Kirby, Anne-Eva Nieuwelink, Bonny W M Kuipers, Anton Kaiser, Pieter C A Bruijnincx, Bert M Weckhuysen

**Affiliations:** [a] Inorganic Chemistry and Catalysis, Debye Institute for Nanomaterials Science, Utrecht UniversityUniversiteitsweg 99, 3584 CG Utrecht (The Netherlands); [b] Van ‘t Hoff Laboratory for Physical and Colloid Chemistry, Department of Chemistry, Faculty of Science, Utrecht UniversityPadualaan 8, 3584 CH Utrecht (The Netherlands); [c] Clariant Competence Center SurfactantsIndustrieparkstrasse 1, 84508 Burgkirchen (Germany)

**Keywords:** calcium, colloids, heterogeneous catalysis, nanostructures, oligomerization

## Abstract

Glycerol is an attractive renewable building block for the synthesis of polyglycerols, which find application in the cosmetic and pharmaceutical industries. The selective etherification of glycerol to higher oligomers was studied in the presence of CaO colloids and the data are compared with those obtained from NaOH and CaO. The materials were prepared by dispersing CaO, CaCO_3_, or Ca(OH)_2_ onto a carbon nanofiber (CNF) support. Colloidal nanoparticles were subsequently dispensed from the CNF into the reaction mixture to give CaO colloids that have a higher activity than equimolar amounts of bulk CaO and NaOH. Optimization of the reaction conditions allowed us to obtain a product with Gardner color number <2, containing no acrolein and minimal cyclic byproducts. The differences in the CaO colloids originating from CNF and bulk CaO were probed using light scattering and conductivity measurements. The results confirmed that the higher activity of the colloids originating from CaO/CNF was due to their more rapid formation and smaller size compared with colloids from bulk CaO. We thus have developed a practical method for the synthesis of polyglycerols containing low amounts of Ca.

## Introduction

**Scheme 1:**
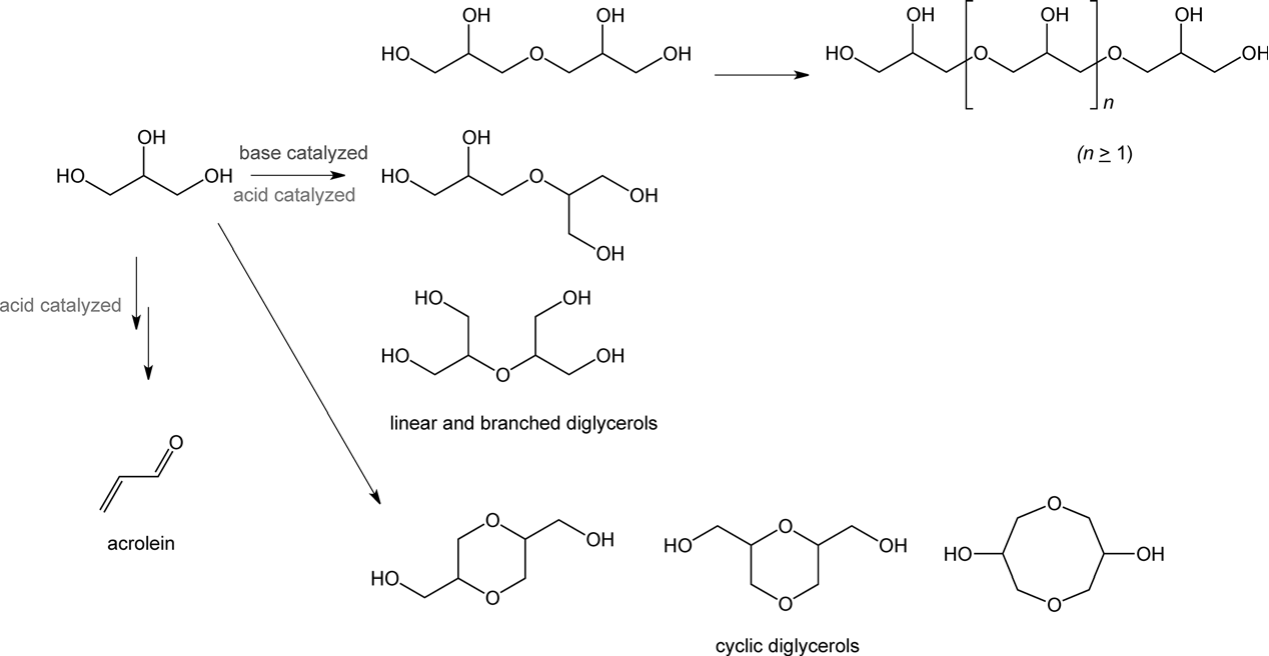
Glycerol oligomerization to the desired open-chain oligomers, undesired cyclic ethers and dehydration to acrolein.

Glycerol is a simple yet functionalized, renewable platform chemical that can be used as a building block for a large variety of commodity chemicals.[1–5] One such valorization route is the direct catalytic etherification of glycerol to polyglycerols. The eventual application of these polyglycerols depends on the (average) degree of oligomerization, with most studies focusing on a low degree of oligomerization, that is, dimerization and trimerization.[6] However, also longer-chain polyglycerols with *n*>1 (Scheme 1) have numerous applications in the textile, food, cosmetic, and pharmaceutical industries. Linear polyglycerols are favored over the branched and cyclic ether byproducts that can also be formed by dehydration and decrease the product quality due to sample coloration and their toxicity. Indeed, for high-end cosmetic and pharmaceutical applications, colorless polyglycerol mixtures are required.

The direct catalytic etherification of glycerol has been studied using homogeneous and heterogeneous acids and bases. Homogeneous acid catalysts, for example, H_2_SO_4_, are recognized as fast but unselective, producing polyglycerols with a high degree of oligomerization, but also giving rise to secondary reactions (e.g., dehydration and oxidation), product coloration, and substantial amounts of toxic acrolein.[7] Heterogeneous acid catalysts such as zeolites, mesoporous aluminosilicates, and acid resins have been studied as well, but are also found to produce many byproducts.[7] Instead, homogeneous bases, such as NaOH or Na_2_CO_3_, are typically used commercially and form the benchmark catalysts for glycerol oligomerization.[5] Although hydroxides are stronger bases than carbonates, the latter typically perform better, as a result of their better solubility in glycerol and the polymeric product at the temperatures of around 260 °C, at which this process is typically run.[8] Homogeneous bases give good conversions, but relatively low selectivity to linear products, although the selectivity is better than with acid catalysis.

Solid heterogeneous bases have also been tested in glycerol etherification. Alkali-modified zeolites and mesoporous solids of the MCM-41 family have shown to be active catalysts in this reaction, although solubility of the catalytic solids and leaching of their constituents into the liquid was observed.[9–11] These catalysts are extensively discussed in two review articles by the groups of Richter and Barrault.^6^,^12^] Recently, the use of mixed magnesium–aluminium oxides has also been reported. Good conversion and selectivities to di- and triglycerol were obtained with limited leaching of the materials into solution being observed, although no detailed information on stability and reusability of these catalysts was given.[13] Notably, the homogeneous and heterogeneous bases reported thus far still have a low activity and selectivity to linear polyglycerols with chain lengths longer than diglycerol. Therefore, there is still a need for improvements in the activity, and, in particular, selectivity to higher polyglycerols, of base catalysts for this important glycerol valorization process.

Our group has previously explored the use of alkaline earth metal oxides as solid base catalysts for this reaction.[14] Glycerol conversion was found to increase in the order BaO>SrO>CaO>MgO, with high selectivities to the desired di- and triglycerol. Improved synthesis of the environmentally benign CaO resulted in a material with a favorable balance of basicity and Lewis acidity, and an activity and selectivity comparable to BaO. Density functional theory (DFT) studies reproduced the catalyst activity order of the alkaline earth metal oxide catalysts found by the experiment. Defect sites, such as edges and steps, were found to play an important role in glycerol dissociation, and corroborated the interplay between basic and Lewis acidic sites.[15] Importantly, an induction period was observed with the alkaline earth metal oxides, indicating that catalyst activation and leaching took place. In the case of CaO, the active species involved may be Ca^2+^ ions in the form of calcium glyceroxide, or Ca(OH)_2_ colloids formed as a result of the partial hydroxylation and fragmentation of the solid CaO material by water formed during the etherification reaction. Evidence showed that colloidal particles of 50–100 nm in size were generated during the reaction and that these colloids were mainly responsible for the very high etherification activity. Relatedly, Barrault et al. analyzed alkaline earth metal oxides after glycerol etherification by XRD and observed the recovered solids to have been completely transformed into the corresponding glyceroxides.[6]

For improved catalysis, better control over colloid formation is required. One way to achieve this is to synthesize them on purpose prior to reaction, providing better control over their structural features, rather than to have them form under reaction conditions. In this light, a somewhat counterintuitive, yet very practical strategy would be to presynthesize the colloids on a support material, as this allows us to regulate the catalyst loading and particle size, to give “drop-in” supported CaO colloids that can be rapidly and controllably dispensed at the onset of the etherification reaction.

Carbon supports, especially carbon nanofibers (CNF), are particularly attractive for this approach as they combine high surface areas with a general, yet tunable chemical inertness.[16] An advantage of using a carbon support is that the interaction between the support and active phase is kept as low as possible, leaving the active species mostly unaffected by the presence of the support. Recently, Frey et al. demonstrated that CaO can be supported on CNF to produce small particles of ≈3 nm, yielding an active and stable solid base catalyst for aldol and transesterification reactions.[17] No significant leaching of calcium was observed, but it should be noted that this reaction was run at relatively low temperatures (**≈**60 °C), meaning that the conditions are much less severe than those used in the glycerol oligomerization process (i.e., 220–260 °C). Indeed, the various supported solid base catalysts reported for reactions such as the aldol condensation mentioned above as well as, for example, transesterification reactions to produce biodiesel are not expected to be stable under the demanding, highly polar, and high-temperature conditions of polyglycerol formation. With the aim of dispensing the supported CaO nanoparticles, we intend to make use of this predicted instability.[18–2020]

To this extent, we explore the synthesis and potential of supported CaO as drop-in colloidal catalysts for the production of higher oligomers of glycerol (*n*>1, Scheme 1), while avoiding the coloration of the product as well as the formation of acrolein and cyclic byproducts. CNF were chosen to stabilize and disperse nanoparticles of CaO. On heating the catalyst in glycerol, CaO is efficiently released from the carbon support into the reaction medium. The advantage of using calcium-based materials for this dispensing strategy is that, given their low cost and toxicity, the calcium species formed can be left in the final product, whereas the CNF can be recovered and reused. The catalytic properties of the dispensed CaO were studied in the etherification of glycerol and compared with industrially employed, homogeneous base catalysts. In addition, different calcium-based materials, that is, Ca(OH)_2_/CNF and CaCO_3_/CNF, were synthesized and investigated as catalysts for this reaction. Experimental parameters were optimized to prepare a linear polyglycerol mixture, with an average chain length of triglycerol and higher, exhibiting a desirable Gardner color (<2). The Gardner color scale grades the color, with a number between 1 and 18, of similarly colored liquids, from pale yellow to red.[21] In addition, we will discuss the nature of the active phase in the reaction mixture based on TEM, XRD, conductivity and light scattering characterization of the CaO/CNF catalysts and the active Ca species formed.

## Results and Discussion

### Catalyst preparation and characterization

**Figure 1:**
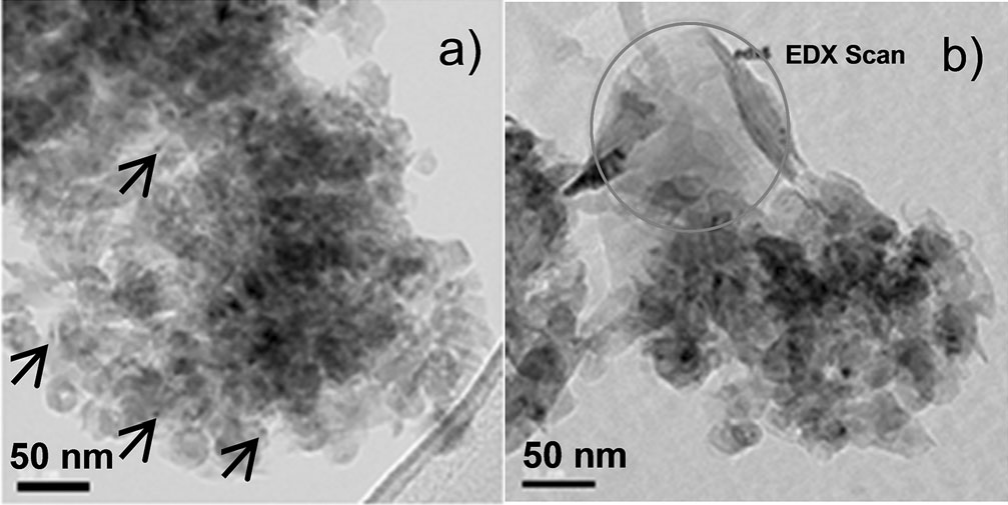
Bright-field TEM image of a) 4.8 % CaO/CNF and b) 14 % CaO/CNF; the circle shows the area that was scanned by TEM-EDX.

CaO/CNF catalysts of various weight loadings (2.5, 4.8, 10, and 14 wt %) were synthesized by incipient wetness impregnation of an aqueous solution of Ca(NO_3_)_2_ on surface-oxidized CNF, followed by a heat treatment at 800 °C in N_2_.[17] Figure 1a shows a representative TEM image of the 4.8 % CaO/CNF catalyst in bright-field mode. At lower-weight loadings of 2.5 and 4.8 % CaO, it was found that CaO nanoparticles of around 5–10 nm in size were dispersed on the CNF support, although the CaO particles are difficult to image due to poor contrast between the CaO and the carbon support. TEM images of the catalysts with higher-weight loadings, 10 and 14 % CaO (Figure 1 b), show that CaO is present as a film or sheet over the support material. Particles were not observed, but TEM-EDX (Figure 1 b, circled) showed that the material was covered with CaO (the Supporting Information, Figure S1).

**Figure 2:**
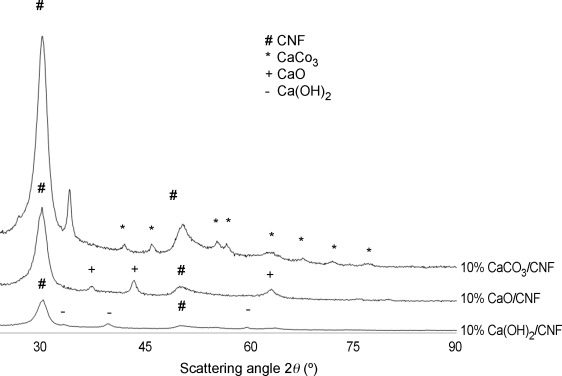
Powder XRD patterns of 10 % CaCO_3_/CNF, 10 % CaO/CNF and 10 % Ca(OH)_2_/CNF.

The crystalline phases present in the supported catalysts were investigated by powder XRD. The XRD pattern of the 10 % CaO/CNF catalyst shows diffraction peaks originating from CaO and CNF (Figure 2). CaO/CNF material of 4.8 and 14 % have similar XRD patterns, with the diffraction pattern of 2.5 % CaO/CNF being dominated by diffraction peaks from CNF. Notably, when the 10 % CaO/CNF catalyst material was exposed to air for 6 h, the XRD analysis showed that the CaO had disappeared and peaks that could be ascribed to Ca(OH)_2_/CNF were now detected. Furthermore, when the heat treatment step of the material synthesis was performed at 400 °C instead of 800 °C, CaCO_3_/CNF XRD peaks were observed, showing that the formation of CaO on the CNF support goes most probably through a carbonate route.[17]

The XRD patterns allowed the crystallite sizes of the supported CaO to be calculated by using the Scherrer Equation (Table 1). The crystallite size of 5–11 nm calculated for the 4.8 % CaO/CNF material is in excellent agreement with the particle size observed by TEM. It was also found that the crystallite size increases with increasing weight loading of CaO onto CNF. As expected, bulk CaO and Ca(OH)_2_ showed much larger crystallite sizes. BET measurements showed the bulk CaO and Ca(OH)_2_ samples to have low specific surface areas, 7–15 m^2^ g^−1^ in accordance with literature, whereas for 14 % CaO/CNF a higher surface area of 124 m^2^ g^−1^ was obtained, which is about half of the surface area of the support (203 m^2^ g^−1^).[22]

**Table 1:** Crystallite size of supported CaO calculated by XRD, specific surface area, and pore volume of the CaO-based catalyst materials under study.

Catalyst	Crystallite size from XRD [nm]	BET [m^2^ g^−1^]	*V*_pore_ [cm^3^ g^−1^]
2.5 % CaO/CNF	n.d.[a]	n.d.	n.d.
4.8 % CaO/CNF	5–11	n.d.	n.d.
10 % CaO/CNF	8–12	n.d.	n.d.
14 % CaO/CNF	12–20	124	0.39
bulk CaO	72–122	7	0.03
10 % Ca(OH)_2_/CNF	9–10	n.d.	n.d.
10 % CaCO_3_/CNF	16	n.d.	n.d.
bulk Ca(OH)_2_	24	15	0.06
CNF	n.d.	203	0.49

[a] Not determined.

### Catalytic activity and selectivity measurements

#### Effect of CaO loading

The series of CaO/CNF catalysts were tested to investigate the impact of CaO loading on the activity in the etherification of glycerol. Reactions were typically performed in a batch glass reaction vessel with 2 wt % CaO/CNF, with a mechanical stirrer, at 220 °C and under an argon flow. A blank reaction with the CNF support material was carried out as well and this experiment reveals a conversion of 10 % to diglycerol after 20 h.

**Figure 3:**
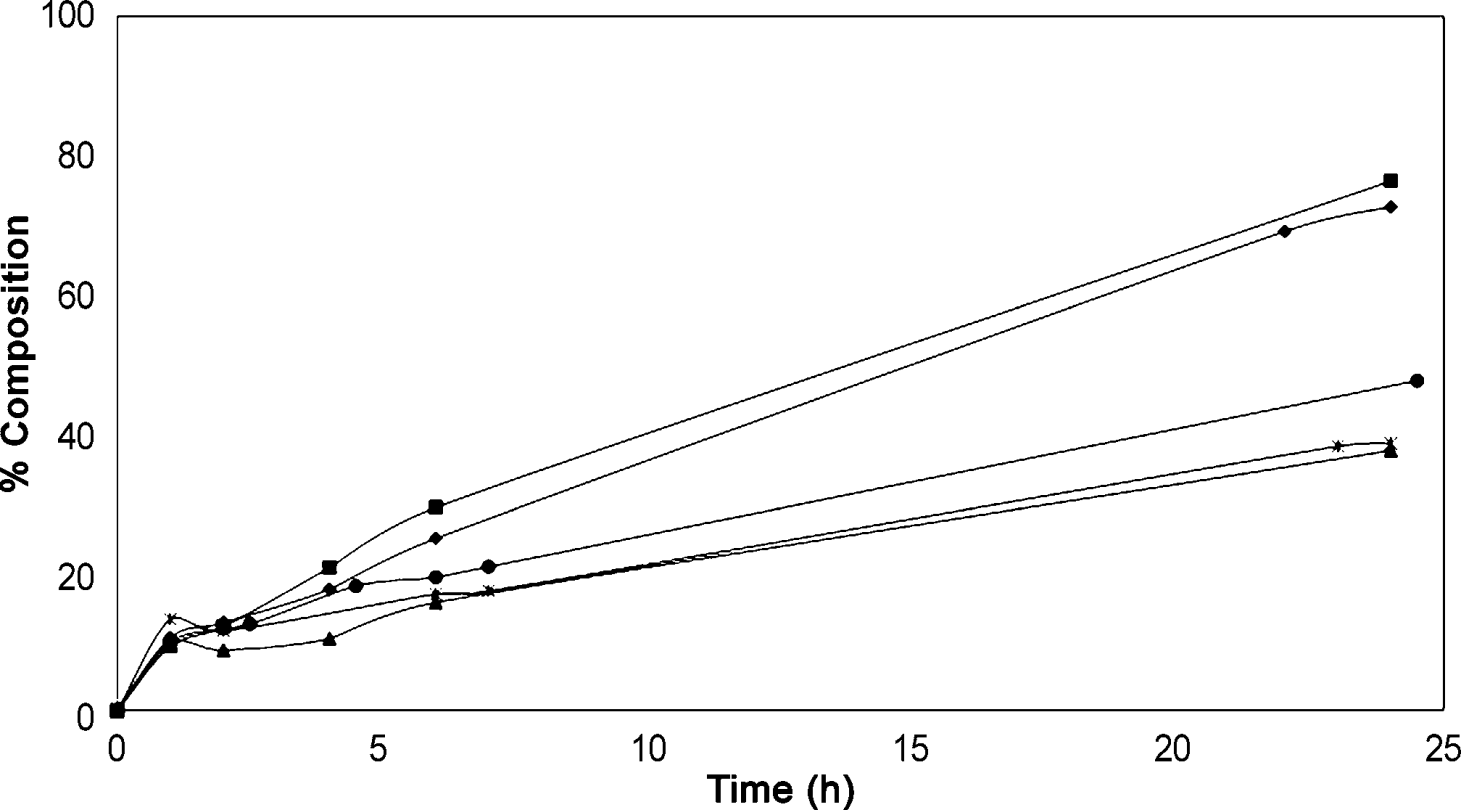
Effect of CaO loading on glycerol conversion as a function of time at 220 °C. Batch reactor with glycerol (100 g, 1.07 mol) and 2 g of catalyst (each CaO/CNF catalyst containing different wt % of CaO). Argon flow with external condensation of water, stirring speed 400 rpm. 14 % CaO/CNF (▪), 10 % CaO/CNF (), 4.8 % CaO/CNF (•), 2.5 % CaO/CNF (▴) and 35.7 mmol CaO (×).

The results are summarized in Table 2, whereas Figure 3 shows the glycerol conversion for the 2.5, 4.8, 10, 14 % CaO/CNF materials and for a reaction run with 35.7 mmol bulk CaO, which is equivalent to 2 g CaO, as a function of time. It can be seen that all catalyst materials are active and that the glycerol conversion increases gradually as the loading of CaO on the CNF support increases. Importantly, the induction period that was previously observed by Ruppert et al. is not observed for the supported CaO colloids, indicating that the colloids are dispensed prior to the onset of the catalysis. The results also show that bulk CaO is much less active than the CaO/CNF nanoparticles, even at a CaO loading that is 7 times higher than the 14 % CaO/CNF material. As was shown in Figure 1, the CaO/CNF with lower loadings of 2.5 and 4.8 % have the CaO present as nanoparticles, whereas the higher loadings of 10 and 14 % CaO have the CaO present as a film/sheet on the support. Dispersing CaO on a support material either as nanoparticles or as thin films greatly increases the CaO surface area, which in turn increases the number of accessible active sites of CaO in glycerol, leading to a higher glycerol conversion.

**Table 2:** Catalytic conversion of glycerol over CaO, Ca(OH)_2_ and differently prepared CaO/CNF catalyst materials, as well as a reference NaOH catalyst.

Catalyst	Ca or Na loading [mmol]	% Conversion (24 h)	Glycerol converted[a] [mmol]	TON[b]
2.5 % CaO/CNF	0.9	37	402	447
4.8 % CaO/CNF	1.7	48	522	307
10 % CaO/CNF	3.6	72	783	218
14 % CaO/CNF	5.0	76	826	165
CaO	35.7	39	424	12
CaO	5.0	43	467	93
NaOH	5.0	50	543	109
Ca(OH)_2_	1.7	43	467	275
Ca(OH)_2_	3.6	55	598	166
Ca(OH)_2_	5.0	49	533	107

[a] Glycerol (mmol) converted per reaction after 24 h.

[b] Glycerol (mmol) converted per mmol metal after 24 h.

Table 2 compares the mmol of glycerol converted per mmol of calcium used per reaction. The 2.5 % CaO/CNF material showed the highest turnover number, with TONs gradually dropping with increased Ca loading, showing that less CaO is available at higher loadings. NaOH was also evaluated as a reference catalyst; 5 mmol of NaOH has a lower glycerol etherification activity when compared with 14 % CaO/CNF (which has equimolar amounts of metal) with glycerol conversions of 50 and 76 % after 24 h, respectively.

**Figure 4:**
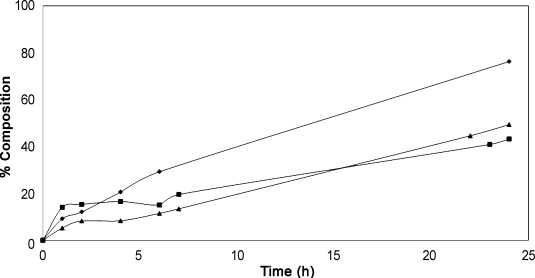
Effect of equimolar amounts of Ca on glycerol conversion as a function of time at 220 °C, for 14 % CaO/CNF (), 5 mmol Ca(OH)_2_ (▴) and 5 mmol CaO (▪). Batch reactor with glycerol (100 g, 1.07 mol) and 5 mmol Ca per reaction. Argon flow with external condensation of water, stirring speed 400 rpm.

The activity of CaO/CNF was compared to bulk CaO and Ca(OH)_2_ at various equimolar Ca loadings. At low loadings of 1.7 mmol Ca (0.16 mol %), the calcium species dispensed by 4.8 % CaO/CNF showed a similar activity to the Ca species formed from 1.7 mmol Ca(OH)_2_, with conversions of 48 and 43 % being obtained after 24 h, respectively. When a higher loading of 5 mmol Ca (0.46 mol %) is used, Ca from 14 % CaO/CNF showed a substantially higher activity than the 5 mmol Ca from CaO or Ca(OH)_2_ (Figure 4). Notably, reactions with 5 and 35.7 mmol of bulk CaO showed very similar activities, that is, conversions of 43 and 39 %, respectively, after 24 h (Table 2). In the reaction with 35.7 mmol CaO, solid material could still be detected in the reaction mixture after 24 h (cf., the complete homogenization of the mixture at 5 mmol catalyst loading) and a calcium glyceroxide phase was observed in the recovered solid (the Supporting Information, Figure S2 a).[6] Notably, the use of purposefully-synthesized calcium diglyceroxide as catalyst for glycerol oligomerization under standard conditions showed an induction period of no less than 7 h, during which time no glycerol conversion was observed (the Supporting Information, Figure S3).

These results indicate that the solubility of (bulk) CaO plays a major role in the catalytic etherification reaction. Indeed, given the limited solubility of CaO in glycerol, only at loadings up to 5 mmol all of the CaO can dissolve in the reaction mixture. We hypothesize therefore that at a Ca loading of 1.7 mmol the reaction is dominated by a homogeneous reaction pathway, for which solubility puts an upper limit to its contribution to catalyst activity. In contrast, at higher loadings of, for example, 5 mmol Ca, there is also a contribution from a heterogeneous reaction pathway due to the presence of colloidal CaO/Ca(OH)_2_ particles. This is in line with previous studies with 35.7 mmol CaO, which showed that after an induction period, Ca(OH)_2_ colloids were produced, which have high catalytic activity.[14] As can be seen from the results presented here, the contribution of the heterogeneous pathway can be optimized by dispersing CaO on CNF, as the presynthesized colloidal nanoparticles are thus efficiently dispensed without an induction period, giving higher activities than equimolar amounts of bulk CaO would in the glycerol etherification reaction.

**Figure 5:**
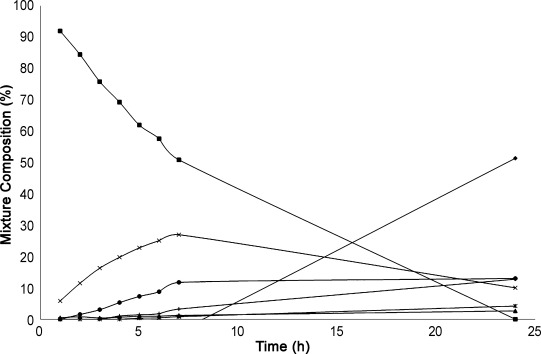
Composition of reaction mixture versus reaction time for CaO/CNF at 220 °C. Batch reactor with glycerol (100 g, 1.07 mol) and 2 wt % catalyst. Argon flow with external condensation of water, stirring speed 400 rpm. Glycerol (▪), cyclic dimers (▴), dimers (×), cyclic trimers (+), trimers (•) tetramers (+) and higher oligomers ().

Figure 5 shows a typical concentration versus time profile for the etherification products. The consumption of glycerol is observed, with a high selectivity of 71 % for diglycerol at 51 % glycerol conversion. With increasing reaction time diglycerol is consumed and higher oligomers of glycerol are formed. Note that oligomers higher than tetraglycerols could not be resolved by our GC method and that the concentration of higher (*n*>2, Scheme 1) oligomers is determined from the balance deficit of product analysis. Importantly, in all cases less than 6 %, selectivity to cyclics was observed.

**Figure 6:**
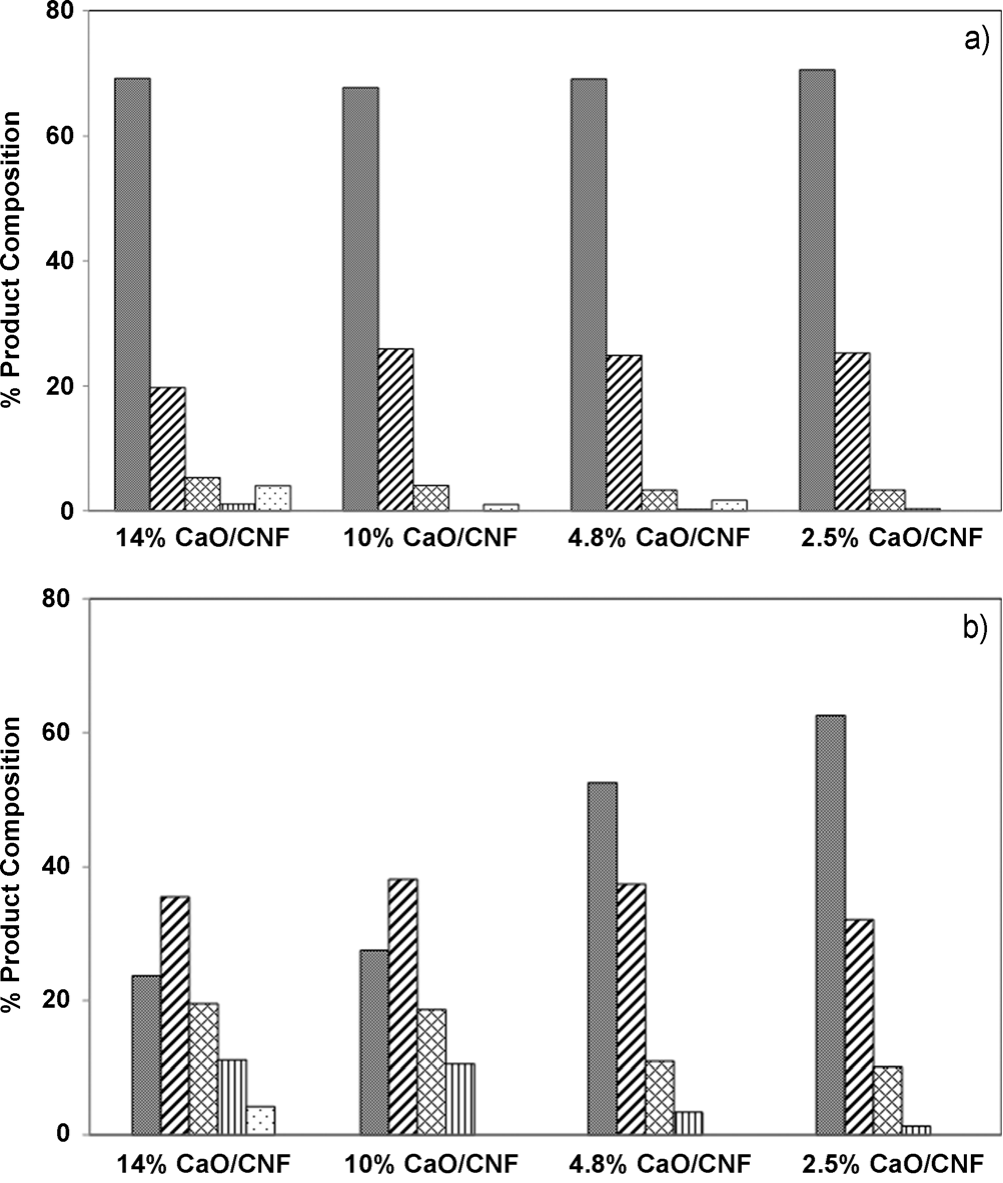
a) Product composition at 30 % conversion; b) Product composition after 24 h reaction time with CaO/CNF catalysts of different weight loading. Batch reactor with glycerol (100 g, 1.07 mol) and 2 wt % catalyst (each CaO/CNF catalyst containing different wt % of CaO). Argon flow with external condensation of water, stirring speed 400 rpm, 220 °C. Glycerol (fine cross hatch), dimers (thick diagonal), trimers (coarse cross), tetramers (vertical lines) and higher oligomers (dots).

The product distributions shown in Figure 6 obtained for the CaO/CNF catalysts of different weight loadings at 30 % conversion are all very similar. After 24 h of reaction time, however, significant differences in product composition are seen. The 10 % CaO/CNF catalyst showed the highest percentage of lower oligomers, that is, dimers and trimers (57 %), whereas 14 % CaO/CNF produced more longer-chain oligomers, containing four or more glycerol units. The GC traces showing the product composition corresponding to the data shown in Figure 6 a are given in Figure S4 (the Supporting Information).

**Figure 7:**
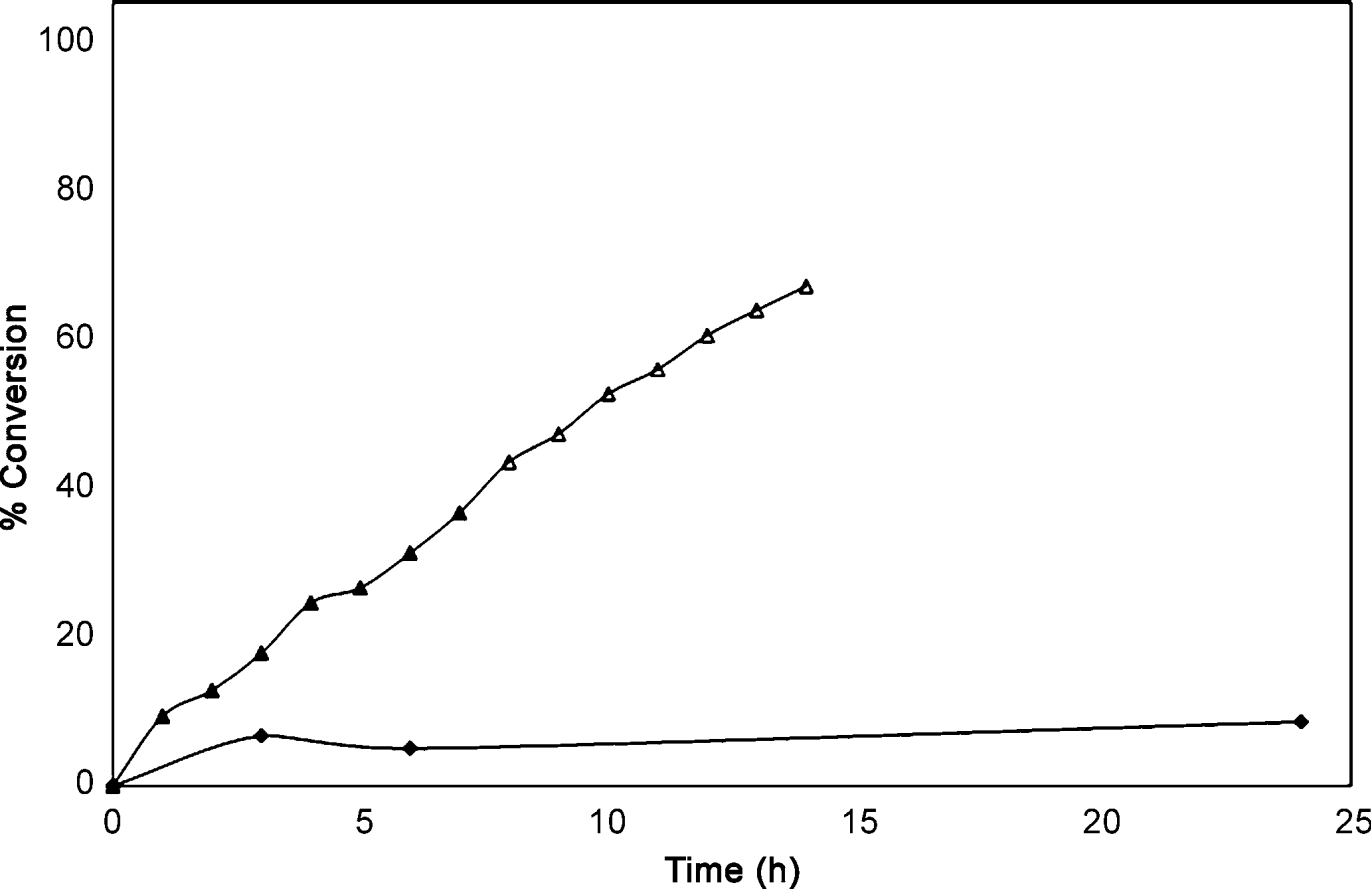
Dispensing experiments of CaO/CNF. 14 % CaO/CNF (▴), spent 14 % CaO/CNF (). The open triangles indicate the conversion after removal of the CNF by hot filtration.

To check the efficiency of the dispersion of CaO into the reaction mixture from the CNF, a hot filtration of the reaction mixture was performed to remove the CNF halfway through the reaction. The data shown in Figure 7 illustrates that the etherification reaction continued at the same high rate, whereas the recovered CNF did not give any significant activity upon reuse. This confirms that all the CaO has been dispensed into the reaction mixture. Atomic absorption spectroscopy (AAS) analysis confirmed that already after 1 h of reaction, 83 % of Ca from the original CaO/CNF material could be found in the liquid phase. XRD analysis of the removed carbon material showed only CNF diffraction peaks (the Supporting information, Figure S2 b).

### Effect of temperature on activity and product coloration

**Figure 8:**
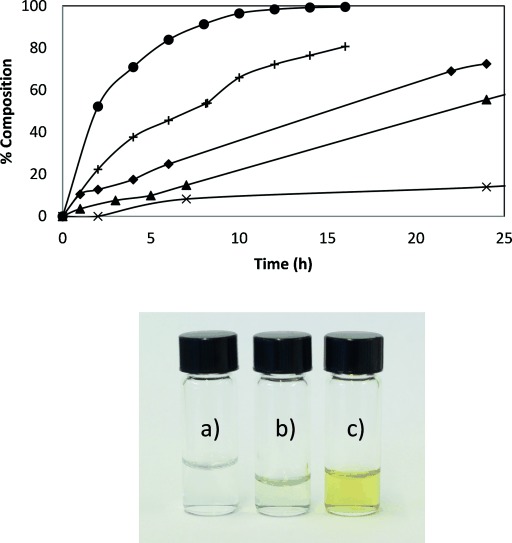
Effect of reaction temperature on glycerol conversion, experiments performed with 10 % CaO/CNF. Reaction carried out at 180 °C (×), 200 °C (▴), 220 °C (), 240 °C (+), 260 °C (•) in a batch reactor with glycerol (100 g, 1.07 mol), and 2 wt % catalyst. Argon flow with external condensation of water, stirring speed 400 rpm. Picture shows product color at 70 % conversion at a) 220 °C, b) 240 °C, and c) 260 °C.

Glycerol etherification is typically carried out at 260 °C, with lower temperatures resulting in a decrease in glycerol conversion.[12] Our group previously reported that lower reaction temperatures (220 °C) also reduce the formation of acrolein and minimize discoloration of the polyglycerol product.[14] We therefore typically perform the glycerol etherification reaction at 220 °C, but to find the optimal temperature for high glycerol conversion, while maintaining the production of a high quality polyglycerol product with *n*>2 (Scheme 1) and Gardner color number <2, we explored a range of reaction temperatures. Figure 8 shows that lowering of the reaction temperatures to 180–200 °C comes at the expense of activity, as shown by the significant decrease in glycerol conversion observed for 10 % CaO/CNF. On increasing the reaction temperature to 240 and 260 °C, the glycerol conversion increases, as expected. Slight discoloration did occur at 240 °C, whereas at 260 °C acrolein and condensation products caused more significant darkening of the product. Importantly, the product mixture obtained after 24 h of reaction time at 220 °C was colorless with *n*=3.5 glycerol units per molecule, as determined from hydroxyl value analysis. At 220 °C, reaction times greater than 24 h give product mixtures with a longer average oligomer chain length, but the coloration also increases with reaction time. If the reaction with 14 % CaO/CNF is, for example, allowed continue to 100 % glycerol conversion, the product mixture is characterized by a slight coloration, corresponding to Gardner color number 1.3, and an average chain length of *n*=4.5 glycerol units per molecule. A reaction temperature of 220 °C thus proved to be optimal for this catalyst system.

#### Influence of the Ca phase

**Figure 9:**
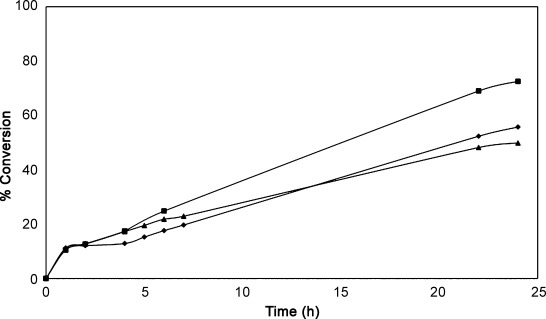
Effect of the type of supported calcium salt on glycerol conversion after a reaction time of 24 h. Batch reactor with glycerol (100 g, 1.07 mol) and 2 wt % catalyst, each catalyst containing 3.6 mmol of Ca. Argon flow with external condensation of water, stirring speed 400 rpm, 220 °C. CaO/CNF (▪), CaCO_3_/CNF () and Ca(OH)_2_/CNF (▴).

As CaO is sensitive to CO_2_ and H_2_O, the phase behavior and reactivity of (supported) CaO is of interest. To investigate this, we also purposefully synthesized Ca(OH)_2_/CNF and CaCO_3_/CNF and compared the performance of these catalysts to CaO/CNF in the etherification of glycerol, all at identical molar loading of 3.6 mmol Ca. Figure 9 shows that CaO/CNF is the most active catalyst, with 73 % conversion after 24 h. There was no difference in selectivity observed between the catalysts. Previous studies with bulk CaO, CaCO_3_ and Ca(OH)_2_ have shown that even though CaO is a stronger base than Ca(OH)_2_ and CaCO_3_, Ca(OH)_2_ is more active than CaO and CaCO_3_ in the etherification of glycerol, a difference that can be attributed to the higher solubility of Ca(OH)_2_ in glycerol and its polymeric products at elevated temperatures.[12] By distributing CaO on CNF this increases the efficiency of dispensing CaO and as a result the catalytic activity. This indicates that the activity of catalysts in glycerol etherification is dependent both on the dispersion of the active species and the base strength of the catalyst.

### CaO stability and related colloidal particle formation

The data clearly show that the catalytic activity of CaO for glycerol etherification can be increased by dispersing the CaO as nanoparticles on CNF. As discussed above, we propose that at a loading of 1.7 mmol CaO, homogeneous catalysis is dominant. At higher loadings of CaO, that is, 5 mmol, heterogeneous catalysis contributes to the reaction, as a result of the presence of colloidal CaO. The efficiency and extent with which the glycerol can be charged with such colloidal catalysts is therefore key to the productivity of the system. Indeed, with 5 mmol CaO, we observe higher catalytic activity when the CaO is dispersed on CNF and dispensed from the CNF into the reaction mixture. We hypothesize that this is due to smaller colloidal particles being rapidly produced from CaO/CNF and immediately contributing to catalysis, whereas larger colloids originating from bulk CaO form over a longer time period as the reaction proceeds and thus do not immediately contribute to the catalytic activity.

CaO dissolution in glycerol involves a complex network of reactions.[23] The harsh temperatures and polar environment necessary for glycerol etherification ensure CaO is converted into different species during the progress of the reaction. As water is produced as a byproduct, Ca(OH)_2_ formation is unavoidable even under argon atmosphere (1) and CaO can react with glycerol to form calcium glyceroxides (2). The active species may also be dissolved Ca^2+^ ions from (3) or (4). The active calcium species may, finally, be present as Ca colloidal nanoparticles. The activity of the soluble Ca^2+^ species from bulk CaO or CaO/CNF is expected to be the same, whereas, the nature of the colloids formed from each source is likely to be different and to produce different activities. To verify these hypotheses we have examined our system by using light scattering techniques and conductivity measurements.

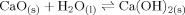
(1)

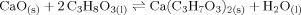
(2)

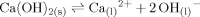
(3)

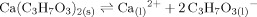
(4)

**Figure 10:**
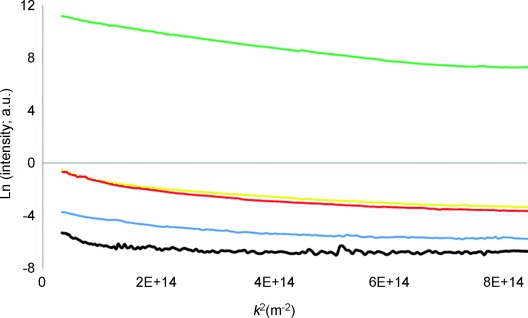
Static light scattering results for 14 % CaO/CNF (red), 4.8 % CaO/CNF (blue), their equivalent Ca molar amounts of 5 mmol Ca(OH)_2_ (green) and 1.7 mmol Ca(OH)_2_ (yellow) and glycerol (black). Samples were taken after 24 h reaction time from a batch reactor with glycerol (100 g, 1.07 mol) and 2 wt % CaO/CNF catalyst (each catalyst containing different wt % of CaO), 5 mmol Ca(OH)_2_ or 1.7 mmol Ca(OH)_2_. Argon flow with external condensation of water, stirring speed 400 rpm, 220 °C.

Light scattering techniques were used to characterize the Ca colloids present. Figure 10 summarizes the static light scattering (SLS) measurements we have performed, plotting the scattered light intensity versus *k*-squared, in which *k* is a measure for the scattering angle.[24] Samples were taken after 24 h from glycerol etherification test reactions carried out with CaO/CNF and Ca(OH)_2_, at two Ca loadings of 5 and 1.7 mmol. In the case of 5 mmol Ca, the scattering intensity of the Ca colloids originating from Ca(OH)_2_ was higher, suggesting the formation of a higher number of small particles or a lower number of larger particles from 5 mmol Ca(OH)_2_ than from 14 % CaO/CNF. The generation of a smaller number of larger colloids from 5 mmol Ca(OH)_2_ can explain the higher activity of 14 % CaO/CNF compared with 5 mmol Ca(OH)_2_ (Figure 4). There was no significant difference in the scattering intensity produced from the colloids of 4.8 % CaO/CNF and 1.7 mmol Ca(OH)_2_. This correlates well with the similar glycerol conversions produced by these two catalysts and confirms that colloids do not play a major role at this Ca loading.

Dynamic light scattering (DLS) was also performed on the same samples that were studied by SLS. DLS can detect nanoparticles and establish a mean radius for an ensemble. The derived count rate (DCR) in Table 3, a measure of the light scattering intensity, shows the same trend as that seen in SLS, with the colloids generated from Ca(OH)_2_ having a higher scattering intensity compared to the colloids generated from CaO/CNF. High polydispersity indices of 0.9 and 0.7 were found for the colloidal particles produced from 14 % CaO/CNF and 4.8 % CaO/CNF, respectively, whereas the 1.7 and 5 mmol Ca(OH)_2_ samples gave a PDI of 1. As PDI values greater than 0.7 indicate that the sample has a very broad size distribution, this precluded further quantification.[25]

**Table 3:** Derived count rates (DCR) from dynamic light scattering measurements and conductivity of product mixtures at various Ca loadings after 24 h reaction time.

Catalyst	Ca [mmol]	DCR [a.u.]	Conductivity [μS cm^−1^]
2.5 % CaO/CNF	0.9	n.d.	124
4.8 % CaO/CNF	1.7	3.9	184
10 % CaO/CNF	3.6	n.d.	337
14 % CaO/CNF	5.0	5.2	423
Ca(OH)_2_	1.7	2.6	197
Ca(OH)_2_	3.6	n.d.	346
Ca(OH)_2_	5.0	8.5	699
Ca(OH)_2_	27.0	n.d.	1160
CaO	5.0	n.d.	485
CaO	35.7	n.d.	1059

**Figure 11:**
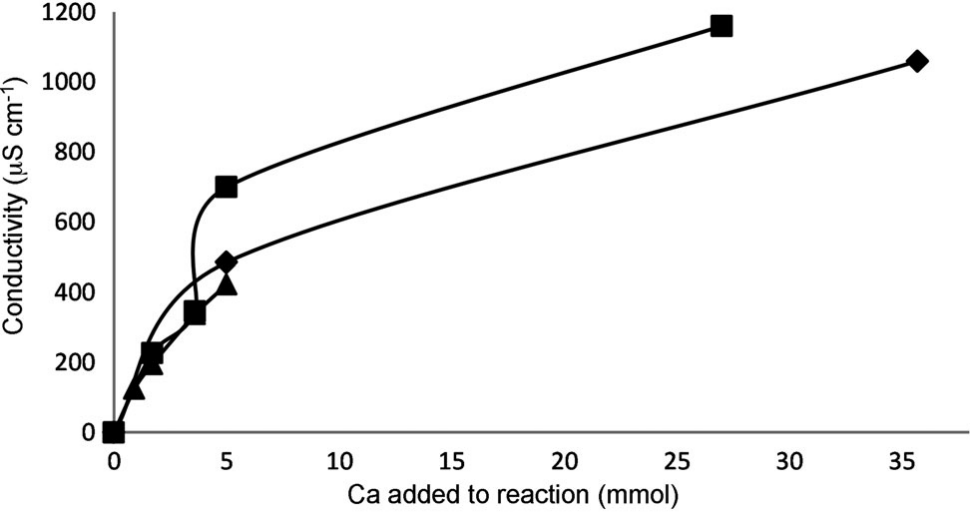
Conductivity of product mixtures at various Ca loadings after a reaction time of 24 h. Samples were taken from a batch reactor with glycerol (100 g, 1.07 mol) and 2 wt % CaO/CNF catalyst (each catalyst containing different wt % of CaO), 1.7, 3.6, 5.0, and 27.0 mmol bulk Ca(OH)_2,_ and 5 and 35.7 mmol CaO. Argon flow with external condensation of water, stirring speed 400 rpm, 220 °C. Ca(OH)_2_ (▪), CaO (), CaO/CNF (▴).

Conductivity measurements were employed to provide further insight into the relative contribution of molecular/homogeneous Ca^2+^ species during glycerol etherification. Figure 11 represents the conductivity (κ) of the polyglycerol reaction mixture versus the amount of Ca added to the reaction. The graph shows that the conductivity increases upon increasing the amount of Ca in the reaction. Similar conductivities are observed with 1.7 mmol of Ca from CaO/CNF and Ca(OH)_2_, 184 and 197 μS cm^−1^, respectively. As these catalysts produce similar activities we can assume that we have mainly homogeneous catalysis occurring at this Ca loading.

Comparing 14 % CaO/CNF and 5 mmol Ca(OH)_2_ or CaO, no remaining solid CaO is observed in the product mixture after reaction and conductivities of 423, 699, and 485 μS cm^−1^ are recorded, respectively. Therefore, there is a lower amount of dissolved Ca^2+^ present with 14 % CaO/CNF, indicating that the increase in activity is not due to an increase in Ca^2+^ in the reaction mixture. We can assume that the rest of the Ca is present in a colloidal form, and that nature of these colloids is responsible for the higher activity of 14 % CaO/CNF. At higher loadings of 2 g CaO (35.7 mmol) and Ca(OH)_2_ (27 mmol), the amount of Ca^2+^ that is soluble in the reaction mixture has reached its limit, because there is solid remaining after reaction for 24 h. As Ca(OH)_2_ is more soluble than CaO it has a slightly higher solubility, yielding conductivities of 1160 and 1059 μS cm^−1^.

We saw from the static light scattering that 14 % CaO/CNF scatters light with a lower intensity than bulk CaO. The conductivity measurements show the increase in activity of 14 % CaO/CNF is not due to an increase in the Ca^2+^ concentration. Therefore, the activity difference between 14 % CaO/CNF and 5 mmol Ca(OH)_2_ or CaO can be explained by smaller Ca colloids being produced from 14 % CaO/CNF giving a higher total surface area of CaO and hence, more active sites.

## Conclusion

We have developed a simple method for the synthesis of highly active Ca colloidal particles for the synthesis of polyglycerols by dispersing CaO as nanoparticles or thin films onto a CNF support. When the CaO colloids were evaluated in the catalytic etherification of glycerol, a polyglycerol product with favorable properties (i.e., colorless, no acrolein, minimal cyclic byproducts, and >3 glycerol units) for applications in the cosmetic industry was obtained. At loadings of 1.7 mmol Ca, CaO/CNF has similar activity in glycerol etherification as equimolar amounts of bulk CaO or Ca(OH)_2_. This is due to the reaction proceeding through a homogeneous pathway at this Ca loading. At Ca loadings of 5 mmol Ca and higher, Ca colloids are present and contribute to catalysis on top of the homogeneous reaction. When these colloids originate from CaO/CNF, higher activities are achieved compared with colloids produced by the fragmentation of bulk CaO. The initial dispersion of CaO on CNF as nanoparticles or films, produces smaller colloids compared to bulk, and thus gives a higher total surface area of CaO, hence more active sites. As a result, the final product contains a lower amount of Ca, but still has all product properties, suitable glycerol chain length, and Gardner color number, required for use in the cosmetic industry. We have thus developed an elegant synthetic method of practical interest to upgrade glycerol to polyglycerol with drop-in CaO-based colloidal catalysts. Due to the use of relatively low amounts of Ca, the polyglycerol products can be used without the elimination of the calcium compounds formed.

## Experimental Section

### CNF growth

A 5 % Ni/SiO_2_ catalyst was prepared by deposition–precipitation synthesis at 90 °C, with nickel nitrate hexahydrate (7.85 g, Acros Organics, 99 %), silica (30 g, Aerosol 300, Degussa), and urea (4.85 g, Acros Organics, 99 %) in water (1.3 L). This material was used as a growth catalyst after calcination (*T*=600 °C) and reduction (*T*=700 °C). CNF were grown from the growth catalyst (5 g) by using syn gas (H_2_/CO/N_2_, 102/266/450 mL min^−1^) at 550 °C and 380 kPa pressure. The fibers were purified by a reflux treatment in KOH (Merck, 85 %, 1 M, *t*=1.5 h) to remove the silica and, after washing, a subsequent reflux treatment with concentrated HNO_3_ (*t*=1.5 h) to remove nickel and to functionalize the fibers. Oxidized carbon nanofibers (typically 30 g) were obtained after the wash.

### Calcium oxide supported on carbon nanofibers

Surface-oxidized CNF (212–425 μm, 2.5 g) were impregnated (0.45, 0.85, 1.80 or 2.50 mmol_metal_ g_catalyst_^−1^) by the incipient wetness method under vacuum with an aqueous solution of calcium nitrate. The catalyst was equilibrated for 1 h at RT, then dried at 120 °C for 12 h in static air. The oxide was obtained by heat treatment of the material at 800 °C (5 °C min^−1^) for 3 h in N_2_. The materials were stored in an Ar atmosphere to avoid exposure to CO_2_ and H_2_O in the air. Ca(OH)_2_/CNF was obtained by flowing water-saturated N_2_ over CaO/CNF for 12 h. CaCO_3_/CNF was obtained as CaO/CNF, except with the heat treatment carried out at 400 °C (5 °C min^−1^) for 3 h in N_2_.

### Characterization

N_2_-physisorption experiments were performed at −196 °C on a Micromeritics Tristar 3000 to determine the specific surface areas of the material after degassing the samples at 180 °C. Powder XRD patterns were obtained using a Bruker-AXS D2 Phaser powder X-ray diffractometer using Co_Kα_ radiation (*λ*=1.789 Å). Measurements we carried out between 10–80 ° 2 *θ* using a step size of 0.08 ° 2 *θ* and a scan speed of 1 s. A PerkinElmer AAS Analyst 200 was used to determine the amount of Ca in ppm in the liquid phase by AAS. The support material was removed by hot filtration before analysis. TEM was performed using a FEI Tecnai 20F. The samples were placed on holy carbon grids and both bright-field and dark-field TEM images were recorded. Static light scattering (SLS) was performed with a FICA 50 setup at a wavelength *λ*_0_=546 nm and a temperature of 25 °C. The samples were diluted with water to decrease the viscosity. During preparation, the mixtures were not protected from dust so the samples were filtered prior to the measurements. Milliporous FP 0.8 μm filters were used. The scattered light intensity was measured as a function of the scattering angle (from 20 to 140).[26] Dynamic light scattering (DLS) was performed by a Malvern Zetasizer Nano using the same samples as for SLS. The hydroxyl value (HV) is the value as determinable by DIN 53240, in which a polyglycerol sample is first acetylated with acetic anhydride in a pyridine solvent, then water is added to hydrolyze the remaining acetic anhydride to produce acetic acid, and the resulting acetic acid is titrated with a titrant up to the endpoint on a Kyoto Electronics Manufacturing titration system or a Metrohm 904 Titrando. Conductivity was measured by using a Seven Excellence Conductivity Meter with a conductivity probe Inlab 731. The samples were diluted with water to decrease the viscosity. The Gardner color was determined by DIN ISO 4630, using a LICO 300 colorimeter. The light-yellow Gardner color numbers (1–8) are based on potassium chloroplatinate solutions, number 9–18 on solutions of ferric chloride, cobaltous chloride, and hydrochloric acid.

### Catalytic testing

Glycerol etherification was carried out in a stirred batch reaction vessel. Glycerol (100 g, Acros Organics, 99+ %) and the catalyst (2 g) were stirred at 220 °C for at least 20 h under argon flow in a five-necked 250 mL flask equipped with a mechanical stirrer (400 rpm) and a Dean–Stark apparatus with a reflux condenser to collect water that was removed from the reaction mixture by the flow of argon gas. Some amounts of glycerol usually also condensed in the Dean–Stark apparatus. Liquid samples were taken periodically.

### Analysis

The reagents and products were analyzed after silylation according to the method of Sweeley et al.[27] Weighed amounts (50–60 mg) of the liquid sample were mixed with pyridine (Acros Organics, 99+ %, 2 cm^3^) and *n*-dodecanol (5 wt % in pyridine) as internal standard in a screw-capped vial (8 cm^3^). After dissolution, hexamethyldisilazane (HMDS, 1.6 cm^3^) and trimethylchlorosilane (TMCS, 0.8 cm^3^) were added and the mixture was heated to 70 °C for 1 h. The solution was injected into a Varian GC equipped with a VF-ms capillary column and an FID detector, in a temperature-programmed mode, ramp 10 °C min^−1^ from 60 to 260 °C, hold 5 min, then ramp 20 °C min^−1^, 300 °C, hold 5 min. Calibrations were performed for glycerol (Acros Organics, 99+ %), diglycerol (Solvay,> 90 %), cyclic dimers (authentic sample synthesized from allyl glycidyl ether[28]), and triglycerol (isolated by silylation and distillation under vacuum from a sample of polyglycerol-4, Solvay). For the calibrations 10, 20, 30, 40, 50, 60 mg of the standards were used to construct the calibration curve. The response factor for tetraglycerol was calculated from polyglycerol-3 (Solvay, 43.3 % triglycerol, 19 % tetraglycerol) and polyglycerol-4 (Solvay, 41.2 % triglycerol, 35.2 % tetraglycerol) and applied to the GC data obtained. The response factor for cyclic trimers was assumed to be the same as for triglycerol. Once the percentage of glycerol, dimers, trimers, tetramers, and cyclics was calculated the missing fraction was assumed to be higher oligomers.
