# Supplementary material for: CaO as Drop-In Colloidal Catalysts for the Synthesis of Higher Polyglycerols
Source: Chemistry. 2015 Feb 13;21(13):5101–9. doi: 10.1002/chem.201405906 (PMC4464545; doi:10.1002/chem.201405906)
Supplement: Supplementary file 1 [file chem0021-5101-sd1.pdf]

CHEMISTRY
A European Journal

Supporting Information

CaO as Drop-In Colloidal Catalysts for the Synthesis of Higher Polyglycerols

Fiona Kirby,[a] Anne-Eva Nieuwelink,[a] Bonny W. M. Kuipers,[b] Anton Kaiser,[c] Pieter C. A. Bruijnincx,*[a] and Bert M. Weckhuysen*[a]

chem_201405906_sm_miscellaneous_information.pdf

Supporting Information

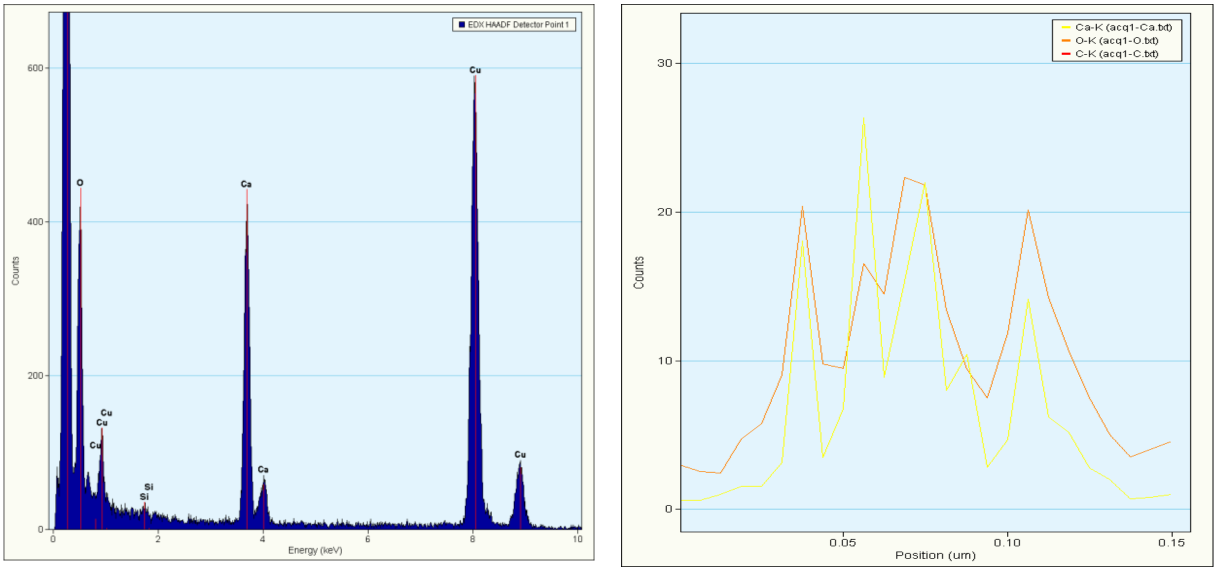

Figure S1: EDX spectrum and line scan analysis of 14% CaO/CNF

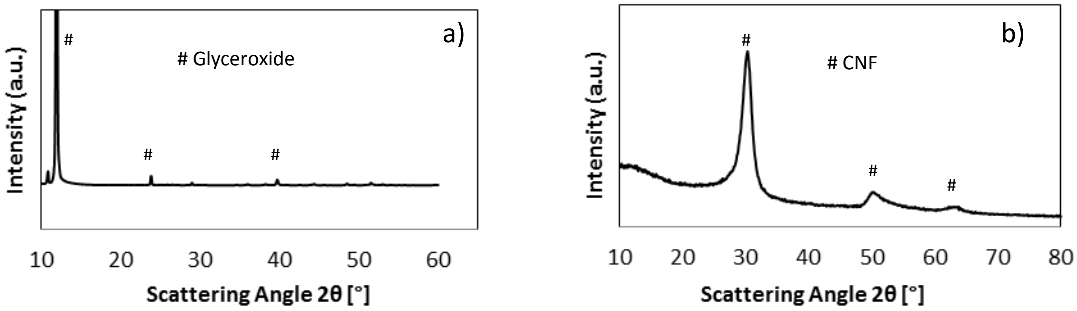

Figure S2: Powder XRD pattern of a) spent CaO and b) spent CaO/CNF.

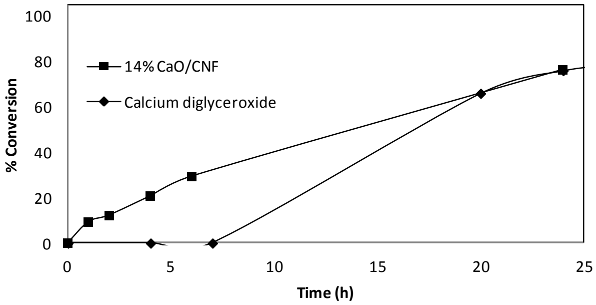

Figure S3: Effect of calcium diglyceroxide at 220 °C and of 14% CaO/CNF on glycerol conversion as a function of time at 220 °C. Batch reactor with glycerol (100 g, 1.07 mol) and 2 g of catalyst. Argon flow with external condensation of water, stirring speed 400 rpm.

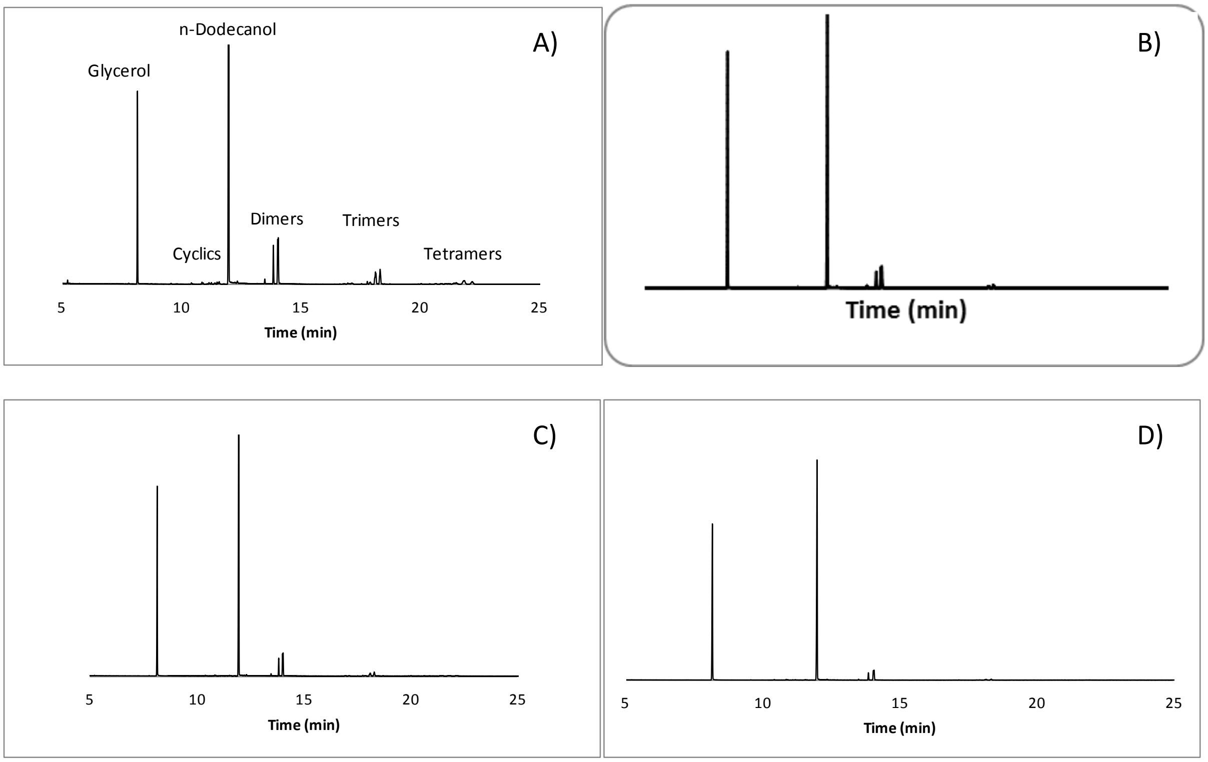

Figure S4: GC traces showing the product composition after 24 h reaction time with CaO/CNF catalysts of different weight loading. Traces correspond to Figure 6A. A) 14% CaO/CNF, B) 10% CaO/CNF, C) 4.8% CaO/CNF and D) 2.5% CaO/CNF.
